# The interplay between DNA damage response and mitochondrial dysfunction in radiotherapy

**DOI:** 10.3389/fonc.2025.1642100

**Published:** 2025-10-20

**Authors:** Shuhua Yang, Yuke Li, Jinlang Zhang, Aihua Shen, Burong Hu, Junfang Yan

**Affiliations:** ^1^ School of Public Health, Wenzhou Medical University, Wenzhou, China; ^2^ Zhejiang Engineering Research Center for Innovation and Application of Intelligent Radiotherapy Technology, The Second Affiliated Hospital of Wenzhou Medical University, Wenzhou, China; ^3^ Wenzhou Key Laboratory of Basic Science and Translational Research of Radiation Oncology, The Second Affiliated Hospital of Wenzhou Medical University, Wenzhou, China

**Keywords:** radiotherapy, DNA damage response, mitochondrial dysfunction, anterograde signaling, retrograde signaling

## Abstract

Radiotherapy plays a crucial role in cancer management by directly eliminating cancer cells, reducing the likelihood of recurrence and metastasis, and preserving the functionality of essential organs. Nonetheless, the radioresistance of cancer cells in radiotherapy poses a significant challenge. The DNA damage response (DDR) serves as a protective mechanism against DNA damage, associating with various intrinsic factors and significantly contributing to radioresistance. Furthermore, the function and status of mitochondria are closely linked to the resistance of cancer cells to radiotherapy. The effects of radiation on nuclear and mitochondrial structures are not independent; they interact through bidirectional signaling pathways to affect cellular radioresistance. This review summarizes and discusses the regulatory mechanisms of DDR and mitochondrial function in radiotherapy from the perspectives of anterograde and retrograde signaling, aiming to provide valuable insights into how cells respond to radiation to determine their fate, and to offer new strategies for precise radiosensitization through the coordinated regulation of nuclear-mitochondrial signaling networks in the future.

## 1 Introduction

Radiotherapy is a cornerstone of cancer treatment and has become an integral component of comprehensive tumor management. By precisely targeting solid tumor cells with high-energy radiation, radiotherapy has been extensively utilized in the treatment of the majority of tumors. Reports indicate that about 70% of cancer patients require radiotherapy at different stages of their illness due to various reasons (1, 2). Advancements in radiotherapy techniques have decreased the treatment-related toxicity, thereby improving long-term patient prognosis. For example, stereotactic body radiotherapy is now considered as the standard therapy for early-stage non-small cell lung cancer patients who are ineligible for surgery, achieving two-year local control rates ranging from 80% to 97% (3).

While radiotherapy takes into account various factors, including Tumor Node Metastasis staging and clinical complications, the biological intricacies of tumors is often neglected. The radioresistance of cancer cells remains a significant challenge in conventional radiotherapy, closely associated with treatment outcomes, prognosis, and tumors recurrence (4). To address this, advances focus on precision radiosensitization. The fundamental principle is to exploit differences in biological characteristics between tumors and normal tissues, thereby enabling selective intervention in specific molecular targets or pathways to maximize tumor cell vulnerability to radiation while sparing healthy tissues (5). Key candidates for radiosensitization include biological processes influencing cellular radiation response, such as DNA damage response (DDR), hypoxia, proliferation and survival pathways, cancer stem cell dynamics, and immune modulation within the tumor microenvironment (5). The DDR involves a complex network of cellular mechanisms crucial for DNA repair and genomic integrity, making it a prime target for radiosensitization (6, 7). Inhibiting hyperactive or synthetically lethal DDR components (e.g., PARP, ATM, ATR, DNA-PK, Wee1) in cancer cells can impair the repair of radiation-induced DNA damage, particularly double-strand breaks (DSB), thereby achieving targeted radiosensitization (7).

Mitochondria, as essential organelles within cells, not only involve in energy metabolism but also mediate various life processes, including cell apoptosis, autophagy, aging, and immune responses (8–10). In radiotherapy, mitochondria are significant targets of ionizing radiation (IR), with their functional integrity profoundly influencing cancer cell responses to IR. Metabolism, biogenesis, and mitophagy are key mechanisms connecting mitochondria to radioresistance (11–13). Consequently, targeting mitochondrial pathways offers a promising approach to enhance radiotherapeutic efficacy and overcome radioresistance.

Importantly, both DDR and mitochondrial radiation effects induced by IR are not single effects, but jointly affect cellular radioresistance through bidirectional signaling between them. Understanding the interplay between DDR and mitochondrial function in radiotherapy is crucial for advancing treatment strategies. By targeting these pathways, it may be possible to enhance the sensitivity of cancer cells to radiation, thereby improving therapeutic outcomes. Therefore, this review seeks to clarify the anterograde and retrograde interactions between nuclear DDR and mitochondria, and proposes novel research perspectives for overcoming radioresistance.

## 2 DDR under ionizing radiation

### 2.1 Types of DNA damages

Genomic DNA is the primary target of radiation in radiotherapy, serving as the key mechanism by which IR eliminates tumor cells (14). Cell death, carcinogenesis, and transformation are all consequences of DNA damages (15). IR induces biological damage, including DNA, through two main mechanisms. The direct mechanism involves radiation energy absorption by atoms, causing ionization (via photoelectric or compton effects) and subsequent chemical bond disruption (16, 17). In contrast, the indirect mechanism involves water radiolysis, producing free radicals (e.g., ·OH and O_2_·^-^) that attack DNA (18). Radiation-induced DNA damage includes various types of damage such as base damage, single-strand breaks (SSB), DSB, with DSBs being the most lethal (19). Radiation quality significantly affects DNA damage complexity and subsequent biological outcomes. Linear energy transfer (LET), which refers to the energy deposited per unit track length, serves as an important indicator of radiation quality. High-LET radiation, such as neutrons and heavy ions, exhibits greater relative biological effectiveness than low-LET radiation, like X-rays and γ-rays. This is due to its dense ionization pattern, resulting in severe, complex DNA damage known as clustered damage, which enhances tumor elimination (20, 21). Although low-LET radiation causes sparse ionization, it still produces significant biological effects through numerous simple lesions (e.g., isolated base damage and SSBs) and an amount of complex clustered damage (22, 23). It is estimated that in clustered damages induced by low-LET radiation (e.g., X-rays), around 1/3 are DSBs, with 70 - 80% of clustered damages containing non-DSB lesions, such as base damage and SSBs (24). These complex lesions, though rare, challenge cellular repair systems and are major contributors to cell death, mutation, and carcinogenesis (22, 23). As LET increases, the complexity of DNA damages also rises (14). Thus, the fate of cells is determined by how they recognize and respond to DNA damage.

### 2.2 Sensing of DNA damage

The DDR is a complex regulatory network in mammalian cells activated by genotoxic stressors. Key events following irradiation include damage sensing by early sensors, recruitment of signaling proteins to initiate cell cycle checkpoints, and engagement of repair factors. Successful repair allows cell survival, while failure leads to cell death (6). Therefore, the fate of the cell depends on the efficacy of DDR, a process systematically elaborated in the review by Rui-Xue Huang et al. (7). This paper will focus on the most lethal form of DNA damage, DSB. The MRN complex (Mre11-Rad50-Nbs1) recognizes DSBs, recruiting and activating ATM through dimer-to-monomer conversion. Activated ATM phosphorylates key effectors, initiating a signal transduction cascade (25). Therefore, ATM activation is the initial “trigger” in the DSB response.

### 2.3 Regulation of cell cycle arrest

IR triggers three main cell cycle arrests: G_1_/S, S, and G_2_/M, where ATM/Chk2 and ATR/Chk1 signaling are the core response pathways (26). Activated ATM can directly or indirectly (via the ATM/Chk2 axis) activate p53, a crucial transcription factor in cell cycle regulation and apoptosis (7, 27). p53 activation leads to the upregulation of p21 CIP1/WAF1, which inhibits Cyclin D-CDK4/6 and Cyclin E-CDK2 complexes, inducing G_1_ arrest (28, 29). The disruption of the G_1_ checkpoint in p53^(−/−)^ cells underscores the critical role of p53 in G_1_/S arrest (30). This arrest allows time for DNA repair, and if repair fail, p53 can initiate apoptosis to prevent mutation accumulation (31, 32). However, there also exists a p53-independent G_1_/S checkpoint mechanism in cells, which is mediated by ATM/CHK2/CDC25C (33). During S phase arrest, ATM slows down or pauses ongoing DNA replication through mechanisms such as ATM/CHK2/CDC25a/CDK2, ATM/Nbs1, preventing replication on damaged DNA templates (7). Additionally, p21, activated in a p53-dependent manner, can also bind to CDK1-cyclin B, blocking G_2_/M progression (34). Research indicates that the defects in the G_2_/M checkpoint mechanism following radiation exposure are linked to mitotic catastrophe, which represents the repair failure. For instance, mutant p53 MEF cells accumulate cyclin B to higher levels and present an increased tendency for mitotic catastrophe while wild-type p53 cells exhibit a markedly lower incidence of such catastrophic events (35). These findings indicate that ATM acts as a central DNA damage sensor, coordinating the cellular response by phosphorylating downstream targets such as Chk2 and p53 to maintain DNA integrity during the cell cycle. Studies have also found that ATM and ATR work synergistically to modulate the G_2_ checkpoint activation under low-dose radiation, achieved via the joint phosphorylation of Chk1. Conversely, in the face of high-dose DNA damage, this cohesive interaction is loosened, with ATM and ATR individually controlling separate components of the cell cycle (36).

### 2.4 DNA repair mechanisms

Given the lethal nature of DSB, their accurate repair is essential. The primary mechanisms orchestrating DSB repair are homologous recombination (HR) and non-homologous end-joining (NHEJ) (6). NHEJ can repair DSBs throughout the cell cycle, but is primarily active during the G_1_ phase. ATM phosphorylates γH2AX, which serves as a scaffold for assembling the DSB repair machinery and stabilizing DNA ends. The Ku70-Ku80 heterodimer recognizes and binds to the broken DNA ends, protecting these ends from being unwound and degraded by cellular nucleases, thus initiating the NHEJ pathway (37–39). Simultaneously, it recruits and activates DNA-PKcs, which phosphorylates downstream repair substrates, triggering a cascade of reactions (6, 7, 40).

Compared to the error-prone NHEJ, HR is relatively more faithful, as it utilizes homologous sequences from the sister chromatid to align the ends of DSBs before ligation. This mechanism is only available during the S and late G_2_ phases of the cell cycle. HR initiation begins with DNA end resection (41). The MRN complex, along with CTIP and BRCA1, initiates the resection of the ends of DSBs (42). Once the ends are resected in a 3’-5’ direction, Ku is released from the DSB ends. The resulting single-stranded DNA can anneal to the unwound sister chromatid. Then Rad51 and BRCA2 form a nucleoprotein filament on the single-stranded DNA, facilitating strand exchange (41).

Clustered damage is classified into single DSBs and non-DSB oxidative clustered DNA lesions (OCDLs) consisting of base lesions and SSB (24). When such lesions are concentrated within a 40 bp genomic segment, they profoundly challenge the DDR system (43). Toshiaki Nakano et al. demonstrated that complex DSBs induced by IR are primarily repaired via the HR during the late S and G_2_ phases. However, this repair is often delayed or unsuccessful (43, 44). Under such extreme genotoxic stress, cells also utilize error-prone backup repair pathways like microhomology-mediated end joining (MMEJ) or alternative end joining (Alt-EJ), which are highly inefficient and mutagenic, frequently causing deletions from several to hundreds of bases (21). OCDLs are primarily repaired through the base excision repair (BER). But the efficiency of this process is influenced by various factors, including inter-lesion distance, spatial orientation, and the specific types of DNA damage (21, 44). For instance, when two lesions (such as apurinic/apyrimidinic sites or SSBs) are in close proximity (within 5 base pairs), the repair efficiency declines drastically (45). Consequently, delayed or failed repair allows damage persistence. During DNA replication, unrepaired OCDLs can convert into more harmful DSBs. In response to these lesions, cells frequently resort to error-prone repair mechanisms rather than accurate HR-mediated repair (21, 44). Notably, there is an overlap among the response and recognition mechanisms for base damages, SSBs and DSBs at multiple DNA damage-inducible sites to maximize the utilization of various pathways. For example, ATR/Chk1 and ATM/Chk2 share the downstream target protein p53, with both Chk2 and Chk1 capable of phosphorylating p53 at Ser 20 (46). Although high-LET radiation exerts strong cytotoxic effects on many tumor cells by inducing complex and irreparable DNA damage, cellular repair mechanisms may still permit survival. This can lead to chromosomal instability and the accumulation of clustered mutations, contributing to acquired radioresistance (21).

### 2.5 Cell death

In addition to the aforementioned mitotic catastrophe, IR can trigger cell death through various mechanisms, such as apoptosis, necrosis, and ferroptosis (47, 48). These modalities of cell demise play a crucial role in cancer treatment as they not only eliminate cancerous cells directly but also bolster therapeutic efficacy by stimulating the immune response and disrupting the tumor microenvironment. Apoptosis, a predominant form of cell death induced by IR, primarily operates via the intrinsic mitochondrial pathway, mediated by Bcl-2 family proteins (48). Resistance to radiotherapy commonly stems from the aberrant regulation of apoptotic elements, characterized by the upregulation of anti-apoptotic molecules (e.g., Bcl-2, Bcl-XL) and the suppression of pro-apoptotic effectors (Bax, Bak) (49–51). Consequently, targeting the Bcl-2 family has emerged as a promising therapeutic strategy to augment radiosensitivity.

Ferroptosis is a type of cell death reliant on iron ions and lipid peroxidation. IR induces lipid peroxidation by elevating reactive oxygen species (ROS) levels and upregulating ACSL4 expression, thereby triggering ferroptosis (52). Moreover, IR can enhance the expression of ferroptosis suppressors like SLC7A11 and GPX4 as a cellular adaptive response (52). Ferroptosis often relies on autophagy. Through RNAi screening, researchers identified several autophagy-related genes as positive regulators of ferroptosis (53). Inhibiting autophagy can impede ferroptosis (53). Studies suggest that post-IR, the interplay between autophagy and ferroptosis may involve lysosomal degradation of mitochondria surrounding lipid droplets, leading to the release of free fatty acids (54).

In certain instances, IR can induce necroptosis, a passive form of cell death characterized by plasma membrane rupture, cell swelling, and leakage of intracellular contents. The core RIPK1/RIPK3/MLKL pathway can be activated by death receptor ligands to release damage-associated molecular patterns (DAMPs) like HMGB1, CRT, ATP, and HSPs, which are highly immunogenic and inflammatory, triggering immunogenic cell death (ICD) (55–58). ICD is a type of cell death that elicits a CD8^+^ T cell-mediated adaptive immune response by releasing DAMPs (57). Ferroptosis is also considered a form of ICD (57). The primary clinical importance of radiotherapy-induced ICD is exemplified by the “abscopal effect” in which localized radiotherapy leads to the regression of distant tumors (57). This phenomenon shifts radiotherapy from a strictly local cytotoxic strategy to a systemic activation of anti-tumor immunity. Stimulating ICD has the potential to augment the efficacy of radiotherapy. For example, adjunctive approaches such as necrosis and ferroptosis inducers can synergistically enhance the anti-tumor effects of radiotherapy (59, 60).

### 2.6 Radiosensitization strategies targeting the DDR

The exploration of DDR pathway targeting to augment radiosensitivity is a prominent focus in cancer radiotherapy research. Encouraging translational evidence from extensive preclinical and early clinical studies highlights the synergistic potential of DDR inhibitors in combination with radiotherapy. Several Phase I clinical trials in solid tumors are currently investigating the safety and initial efficacy of the ATM inhibitor AZD139 combined with radiotherapy for patients with gliomas, intracranial malignancies, soft tissue sarcomas, and pulmonary tumors (ClinicalTrials.gov, data up to September 2025). Early clinical assessments of the ATR inhibitor Berzosertib (VX-970) administered concurrently with radiotherapy for esophageal carcinoma have demonstrated manageable toxicity profiles and promising antitumor responses (61). Noteworthy DDR protein inhibitors include Adavosertib (Wee1 inhibitor), Prexasertib (Chk1 inhibitor), and PARP inhibitors such as Olaparib, Niraparib, and Rucaparib, collectively constituting a radiosensitizing drug regimen targeting the DDR pathway (62–66).

Many cancer cells harbor intrinsic mutations in key DDR genes like BRCA1/2, ATM, and p53, rendering them reliant on alternative DNA repair mechanisms for survival. Exploiting this dependency presents a unique opportunity for synthetic lethal interventions. By concurrently targeting two DDR pathways engaged in a synthetically lethal relationship, tumor cells can be selectively eradicated while preserving normal tissues (67). For example, tumors deficient in ATM function display increased sensitivity to ATR inhibition, leading to a potent synthetic lethal outcome (68). This approach synergistically enhances the efficacy of radiotherapy, as radiation-induced DNA damage, including SSBs and DSBs, intensifies tumor cells’ reliance on DDR signaling. Capitalizing on synthetic lethality with precise inhibitors to disrupt crucial backup repair pathways enables precise and devastating targeting of tumors bearing specific genetic deficiencies. The synthetic lethality between BRCA1/2 mutations and PARP inhibition is one of the most successful and classic precision medicine strategies in contemporary cancer therapy (69). Ongoing research continues to unveil numerous exploitable synthetic lethal interactions within the DDR network for enhancing radiosensitivity. Notably, mutations in p53 have been associated with synthetic lethal relationships involving various DDR components, such as ATR, CHK1, MK2, Wee1, and DNA-PK (70). For instances, recent studies using TP53-mutant medulloblastoma models have demonstrated that combining a DNA-PK inhibitor (Peposertib) with radiotherapy results in significant synergistic antitumor effects and markedly extended survival *in vivo* (71). While the DDR synthetic lethality strategy may encounter challenges like drug resistance from secondary mutations and reactivation of alternative pathways, it remains promising for improving the efficacy of radiotherapy (72). Additionally, it offers valuable insights for investigating alternative synthetic lethal pathways. Understanding the interplay between DDR signal transduction and mitochondrial function in radiosensitivity, for instance, could unveil novel therapeutic approaches for synthetic lethality-based interventions.

## 3 Anterograde signaling from DDR to mitochondria after ionizing radiation

Mitochondria, characterized by their distinctive double-membrane configuration, represent a critical cellular target for IR. IR can instigate a series of intricate alterations in mitochondrial DNA (mtDNA), ROS, and energy metabolism, which in turn dictate distinct cellular outcomes (73–75). The alterations in mitochondrial induced by IR are linked to the quality of the radiation, specifically the LET. Low-LET radiation typically elicits adaptive responses of mitochondria, such as increasing mtDNA copy number, upregulating mitochondrial synthesis, and elevating energy metabolism to compensate for mitochondrial damage (76). Conversely, high-LET radiation tends to induce mtDNA damage, mutations, ROS generation, collectively leading to a pro-apoptotic cell fate (77–79).

Cellular defense against radiation-induced mitochondrial damage involves the activation of mitochondrial quality control system. Mitochondrial biogenesis, mitophagy, and mitochondrial dynamics are critical stages in the monitoring of mitochondrial quality. These processes are orchestrated to preserve mitochondrial stability and rejuvenate the function of impaired mitochondria (80). A dynamic feedback loop interconnects damage and quality control, where successful quality control promotes functional recovery, while its failure leads to mitochondrial dysfunction, manifested as diminished ATP synthesis, elevated ROS levels, dysregulated calcium ion (Ca²^+^) balance, mtDNA mutation accumulation, loss of membrane potential, and abnormal mitochondrial permeability transition pore (mPTP) opening (81). This dysfunction escalates the strain on and disruption of quality control mechanisms, exacerbating functional impairment. This self-reinforcing vicious cycle is a key driver of progressive cellular impairment in aging and numerous diseases (82, 83). In the following sections, we will focus on changes in mitochondrial biogenesis, mitophagy, fission, and fusion induced by IR, and analyze their integration with DDR pathway signaling.

### 3.1 Changes in mitochondrial quality control

#### 3.1.1 Mitochondrial biogenesis

**Figure 1:**
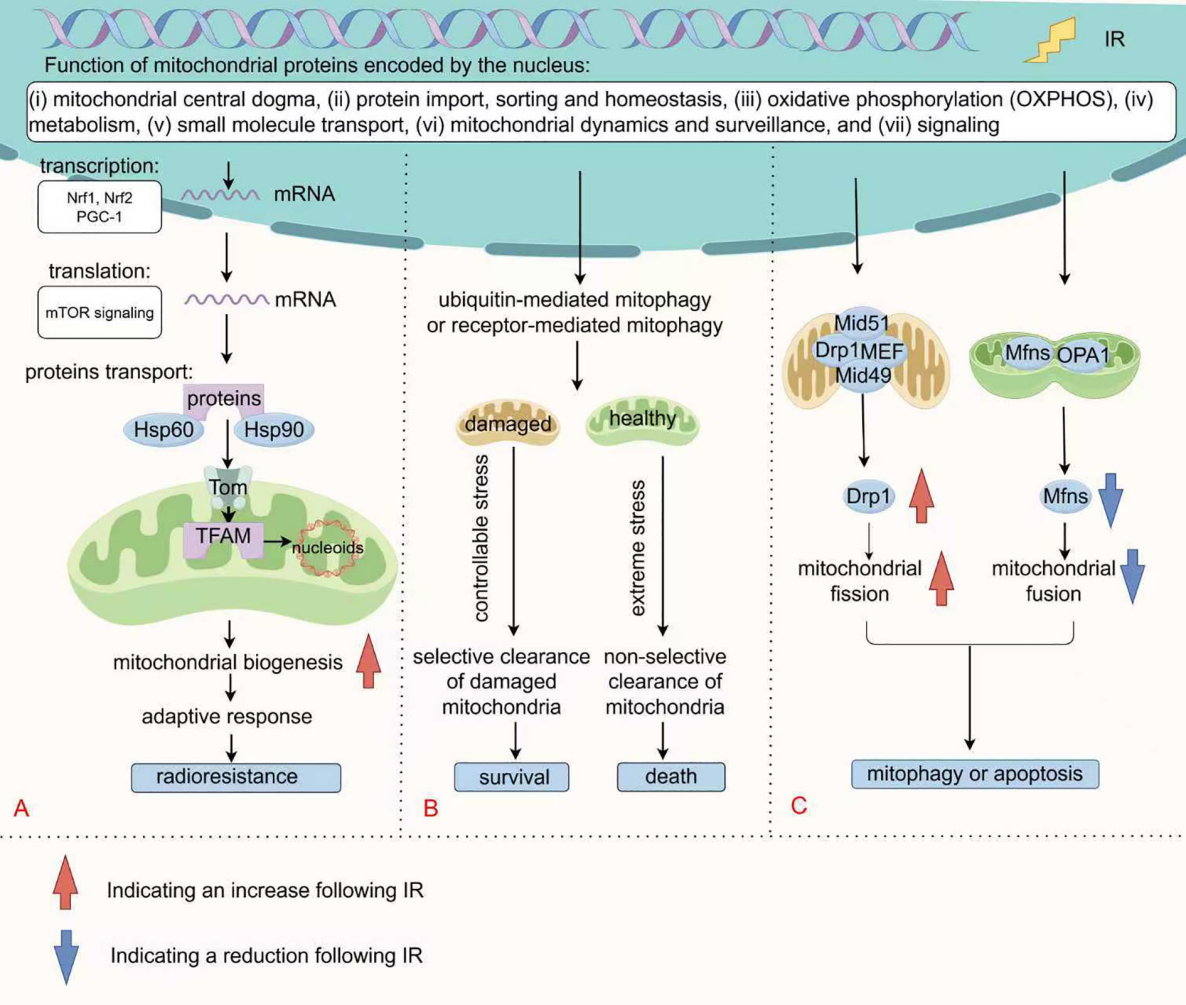
Changes in mitochondrial quality control following IR. **(A)** Mitochondrial biogenesis requires the synchronized regulation of mtDNA and nuclear genome. Nuclear-encoded factors specifically mediate mtDNA replication and transcription, with PGC-1α and Nrfs regulating the transcription of most of these genes. Following mTOR signaling-mediated translation, they enter the mitochondria via the TOM complex with the assistance of molecular chaperones for further folding and assembly. **(B)** Mitophagy selectively eliminates mitochondria through ubiquitin-mediated pathway or receptor-mediated pathway. Under mild stress conditions, moderate mitophagy supports cell survival by clearing dysfunctional mitochondria. Conversely, under severe stress conditions, excessive activation or non-selective mitophagy promotes cell death. **(C)** Mitochondrial fission is mediated by the Drp1 protein and its receptors (such as MFF, Mid49/51), which promote membrane constriction through oligomerization. Fusion is coordinated by Mfn1/2 and OPA1, maintaining functional integrity through content exchange. IR disrupts mitochondrial dynamics by Drp1 (Ser 616/Ser 637) phosphorylation and MFN downregulation, leading to fragmentation of mitochondria.

Mitochondrial biogenesis is the process by which cells respond to extracellular demands by increasing the number and size of mitochondria, which is jointly regulated by mtDNA and the nuclear genome. According to the mitochondrial proteome database curated by Vamsi Mootha’s group at Harvard/Broad Institute, the 1,136 human mitochondrial genes are classified into seven categories: (i) mitochondrial central dogma, (ii) metabolism, (iii) oxidative phosphorylation (OXPHOS), (iv) protein import, sorting and homeostasis, (v) mitochondrial dynamics and surveillance, (vi) small molecule transport, and (vii) signaling (**Figure 1A**) (84). mtDNA is a double-stranded DNA molecule (∼16,569 bp) comprising heavy and light chains that encode 13 peptides integral to OXPHOS complexes, along with 22 tRNAs and 2 rRNAs (85). Its replication and transcription are crucial for mitochondrial biogenesis and are governed by distinct nuclear-encoded factors. Transcription begins at two primary promoters in the D-loop region: the heavy-strand promoter (HSP) and the light-strand promoter (LSP). The single-subunit mitochondrial RNA polymerase (POLRMT), along with transcription factor A (TFAM) and transcription factor B2 (TFB2M), binds these promoters to generate long polycistronic transcripts that nearly cover the entire heavy or light strand. These primary transcripts are then processed by enzymes like RNase P to release individual mRNAs, tRNAs, and rRNAs (86). Importantly, TFAM not only regulates transcription but also compacts mtDNA into nucleoprotein complexes called nucleoids, protecting the genome from degradation through DNA bending and condensation (87, 88).

Mitochondrial DNA replication predominantly follows the canonical strand-asymmetric mechanism (86). DNA polymerase gamma (POLγ) catalyzes synthesis, and mutations in this enzyme can lead to severe progressive diseases (89). The helicase Twinkle unwinds duplex mtDNA using ATP hydrolysis, providing the single-strand template needed by POLγ (90). Concurrently, mtSSB stabilizes exposed single-stranded regions, preventing degradation or secondary structure formation, and significantly enhances the processivity of both Twinkle and POLγ, serving as a crucial regulator of replisome function (86, 91).

In fact, over 99% of mitochondrial proteins are encoded by the nucleus. Transcriptional regulation of nuclear genes for mitochondrial, such as respiratory chain subunits, heme biosynthetic enzymes, mtDNA replication/transcription machinery, and protein import systems, is governed by nuclear respiratory factors (Nrf1 and Nrf2), in concert with the coactivator PGC-1 (92, 93). PGC-1α is broadly recognized as the major regulatory factor in the process of mitochondrial biogenesis (94). Moreover, the translation of mitochondria-related proteins is regulated by the mTOR signaling(to be discussed later) (95, 96). These precursor proteins in the cytoplasm are transported into the mitochondria through translocase outer membrane (TOM) (97). Protein transport, folding and assembly are facilitated by chaperones such as Hsp60 and Hsp90 (98–100). These nucleus-to-mitochondria signaling pathways, known as anterograde signaling, are the primary determinants of mitochondrial biogenesis (**Figure 1A**).

Studies have revealed that tumor cells possess the ability to adapt to radiation-induced DNA damages by boosting mitochondrial biogenesis, which increases DNA copy number and influences radiotherapy response (101). For example, 3.5 MeV α-particle irradiation activates the PI3K/Akt pathway, regulating the expression of TFAM in A549 cells, thereby raising mtDNA copy number and COX catalytic activity (102). Similar phenomena have been observed in normal cells. Eun Ju Kim and colleagues found that IR elevates transcription of glucose transporter genes (Glut1 and Glut4), and mitochondrial biogenesis genes (PGC-1 and CPT-1) in mouse skeletal muscle C2C12 myotubes. This upregulation is accompanied by increasing in mtDNA copy number and ND2 levels, thereby enhancing its oxidative metabolism and reducing glycolytic capacity (103). Since effective DNA repair DDR requires significant ATP and nucleotide precursors, post-irradiation mitochondrial biogenesis provide critical energy for DNA repair (104–107). This metabolic adaptation supports tumor cell survival under IR, contributing to radioresistance.

Therefore, the mitochondrial biogenesis regulators PGC-1α, Nrfs, and TFAM constitute actionable targets for radiosensitization. For example, radiation activates CD105/BMP signaling, which upregulates SIRT1, stabilizes p53, and stimulates PGC-1α-driven mitochondrial biogenesis, resulting in radioresistance. Inhibiting this pathway with the monoclonal antibody TRC105 enhances the efficacy of radiotherapy through synthetic lethality (108). Meanwhile, pharmacological inhibition of Nrf2 with clobetasol propionate (CP) potently sensitizes Keap1-mutant NSCLC A549 cells to irradiation by inducing mitochondrial dysfunction and ferroptosis. Critically, CP has limited impact on normal lung fibroblasts, indicating a favorable therapeutic window. Additionally, CP-mediated Nrf2 inhibition attenuates DNA repair, highlighting the interplay between mitochondrial function and DDR (109). Likewise, TFAM depletion increases ROS production and triggers mitochondrial retrograde signaling, facilitating p53-MDM2 interaction and p53 degradation, thereby enhancing radiosensitivity (110).

#### 3.1.2 Autophagy and mitophagy

Autophagy (referring to macroautophagy here) is a conserved intracellular degradation process in which double-membraned autophagosomes engulf cellular material, such as damaged organelles, excess proteins, or pathogens, and deliver it to lysosomes for breakdown and recycling, thereby preserving cellular homeostasis. This essential process is governed by conserved regulators, including the ULK complex, autophagy-related (ATG) genes, and the microtubule-associated protein LC3 (111). Mitophagy, a specialized form of autophagy, selectively eliminates damaged or dysfunctional mitochondria via ubiquitin-mediated pathway or receptor-mediated pathway. Ubiquitin-mediated mitophagy is controlled by PINK/Parkin or alternative ubiquitination pathways, including the E3 ubiquitin ligase MUL1, while receptor-mediated mitophagy involves the participation of receptors like BNIP3, FUNDC1, and lipid-binding receptors (112). As depicted in **Figure 1B**, depending on the cellular context and the degree of DNA damage, mitophagy can elicit diverse cellular outcomes. It functions as a vital survival mechanism under stressful conditions, facilitating tumor cell viability as a protective mechanism. For instance, tumor cells employ mitophagy to eliminate injuries induced by ROS, thereby causing radioresistance (113). In response to severe mitochondrial damage, mitophagy tends to induce cell death through extensive self-digestion (54, 114). Heng Zhou and his colleagues have reported that carbon ions can trigger mitophagy to facilitate ferroptosis via increased lipid peroxidation (54).

**Figure 2:**
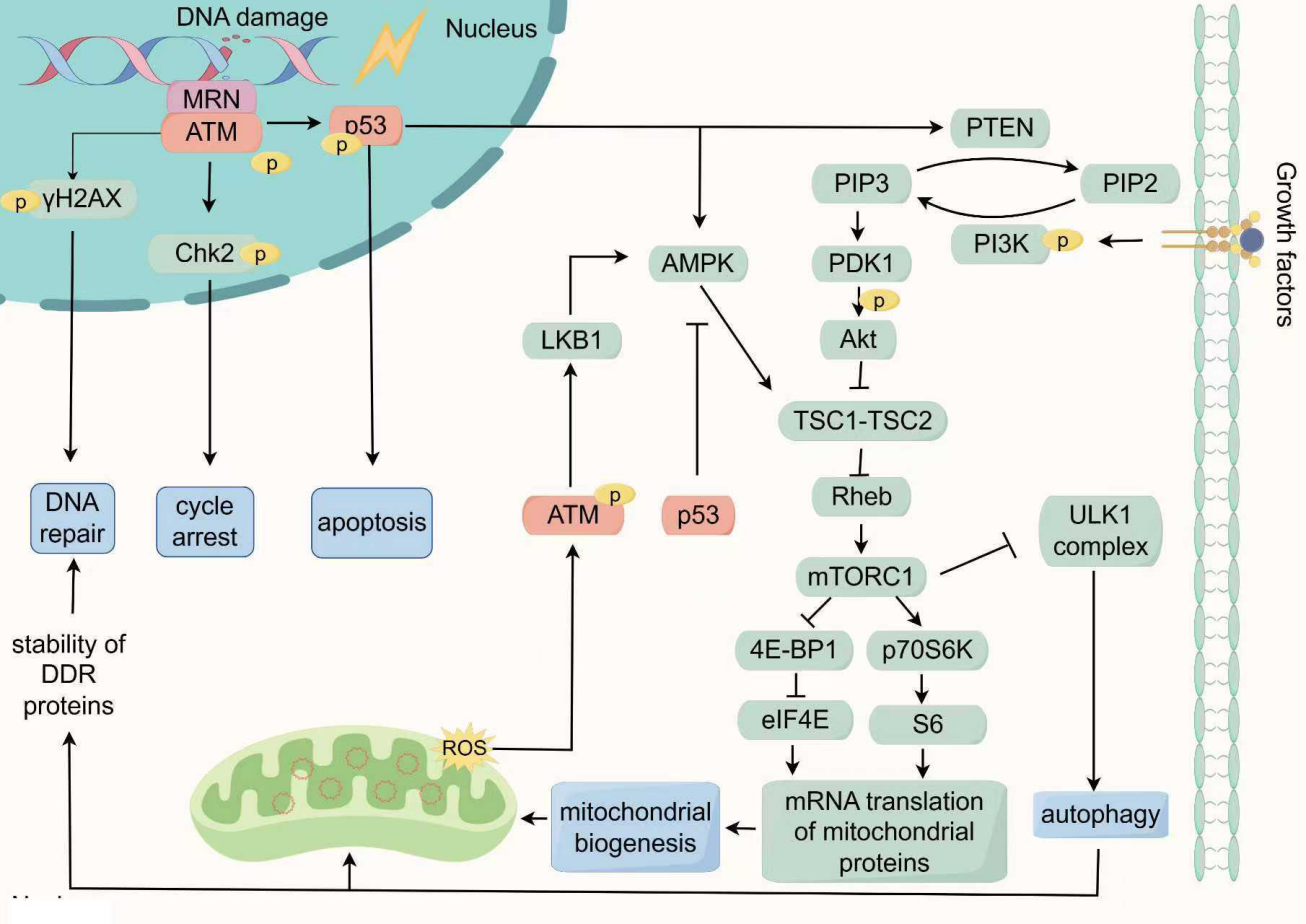
The key response network of DDR in regulating mitochondrial function: ATM/p53/mTOR signaling. In the DDR, the MRN complex recognizes DSBs and recruit ATM to promote autophosphorylation of ATM. Subsequently, Activated ATM phosphorylates downstream target proteins such as p53, Chk2, and γH2AX to regulate cell cycle arrest and DNA repair in the nucleus. The phosphorylation of p53 can activate the transcription and expression of pro-apoptotic genes, triggering cell apoptosis. Additionally, p53 can also trigger autophagy by activating AMPK or inducing PTEN transcription to suppress the mTOR pathway. In the cytoplasm, ROS can activate ATM by a novel mechanism which is based on intermolecular disulfide bonds. This phosphorylation event regulates the LKB1/AMPK pathway, leading to inhibition of mTORC1 and the initiation of autophagy. Conversely, cytoplasmic p53 inhibits the activation of AMPK, thereby suppressing autophagy. The regulation of mTORC1 activity involves the antagonistic effects of AMPK and PI3K/Akt, with PI3K/Akt acts as a positive regulator. The activation of mTORC1 further stimulates eIF4E and S6, promoting mitochondrial biogenesis by modulating the translation of nuclear-encoded mitochondrial-related proteins. Simultaneously, autophagy positively regulates the DNA repair system by maintaining the synthesis and degradation balance of DDR proteins. Consequently, the ATM/p53/mTOR signaling network responds to DNA damage by orchestrating the cell cycle, DNA repair mechanisms, apoptosis, autophagy, and mitochondrial biogenesis, all of which are essential for maintaining cellular homeostasis and governing cell fate.

Increasing evidences indicate that autophagy can regulate DDR through maintaining the balance between synthesis and degradation of DDR proteins (**Figure 2**). In mice deficient in ATG7, the inhibition of autophagy increased proteasome activity, resulting in enhanced degradation of Chk1 and great reduce of HR repair. This impairment in HR repair was associated with higher rates of apoptosis and micronuclei formation (115, 116). Additionally, autophagy induced by rapamycin, an mTOR inhibitor, was found to mitigate nuclear radiation damage by preserving DNA repair proteins such as DNA ligase 4, Ku80, XRCC4, and BRCA1 (117). These findings suggests that autophagy positively modulates the DNA repair system, particularly the HR pathway, providing a critical time window for DNA damage processing.

Therefore, under physiological conditions or moderate stress, autophagy serves a cytoprotective role. However, under pathological or severe stress, uncontrolled autophagy induces massive non-selective elimination of mitochondria, surpassing the capacity for functional recycling and ultimately causing cell death (118). Furthermore, research indicates that caspases, as core executioners of apoptosis, proteolytically process selected ATG proteins, resulting in functional alteration and a transition from autophagic survival to death signaling (119). This transition from pro-survival to pro-death is not due to change in the fundamental purpose of autophagy, but rather results from its hyperactivation, dysfunction, or collaboration with other cell death pathways. Thus, radiosensitization strategies seek for disrupting this protective balance, for instance by inhibiting early protective autophagy to prevent cancer cell self-repair, or by enhancing radiation-induced stress to drive excessive autophagy and trigger cell death.

#### 3.1.3 Mitochondrial fission and fusion

Mitochondrial fission and fusion, collectively referred to as mitochondrial dynamics, are essential for maintaining mitochondrial morphology, functionality, and cellular health. As shown in the **Figure 1C**, mitochondrial fission is primarily regulated by Drp1, which is recruited to the outer mitochondrial membrane, where it oligomerizes to constrict and divide the mitochondria (120). This recruitment is mediated by specific receptor proteins: MFF serves as the primary anchor, while Mid49 and Mid51 assist in Drp1 assembly. In mammals, Fis1 appears to play a minor role and is not required for fission (121). Once recruited, Drp1 forms GTP-hydrolyzing helical structures that narrow the mitochondria, with final scission mediated by Dyn2 (122). Recent studies have highlighted the role of post-translational modifications, such as phosphorylation and acetylation, in modulating Drp1 activity (120, 123). While fission aids mitochondrial turnover and the removal of damaged components via mitophagy, overactivation can lead to pathological fragmentation and dysfunction. For instance, oxidative stress can enhance Drp1 activity through JNK-mediated phosphorylation, leading to increased mitochondrial fragmentation and dysfunction (124).

Mitochondrial fusion serves to maintain mitochondrial function by enabling the exchange of mitochondrial contents. This process is chiefly facilitated by mitofusins (Mfn1 and Mfn2) and OPA1 (125). Mitofusins drive membrane fusion through GTPase-dependent tethering and oligomerization, forming intermitochondrial bridges that fuse following conformational changes (126). OPA1, present in long (L-OPA1) and short (S-OPA1) isoforms, governs inner membrane fusion. The balance between these isoforms is essential, with L-OPA1 directly involved in forming the fusion pore (123). Fusion helps to mitigate the effects of damaged mitochondria by facilitating the sharing of resources and compensating for dysfunctional units within the mitochondrial network.

Radiation often perturbs the balance of fission-fusion, promoting excessive fission. Research has demonstrated that γ-rays can induce a dose-dependent increase in mitochondrial fission, marked by elevated level of Ser 616/Ser 637 phosphorylation of Drp1 and decreased MFN expression, leading to greater mitochondrial fragmentation (127). Continuous mitochondrial fusion and fission constitute an important mechanism for intracellular signal encoding and communication. A fused mitochondrial morphology signifies a “healthy, energy-sufficient” state and favors nuclear signaling that supports survival and homeostasis (128). In contrast, fragmented mitochondria often represent a “stressed or injured” state and initiate signals for mitophagy or apoptosis (129, 130). For instance, Yujia Li et al. demonstrated that Drp1 overexpression causes mitochondrial dysfunction, releasing mtDNA into the cytoplasm, activating the cGAS-STING pathway, and promoting autophagy and tumor growth (131). Another study showed that silica nanoparticles induce cardiomyocyte apoptosis via PKA-DRP1-mediated mitochondrial fission (132). In this context, radiation acts as molecular switch that promotes a fragmentation-dominant state through Drp1 activation and MFN inhibition, thereby translating radiation stress into a morphological signal that instructs cell fate decisions between death and survival (**Figure 1C**). Evidences demonstrate that Drp1 inhibition decreases cellular radiosensitivity (133, 134). Thus, actively promoting radiation-triggered mitochondrial fragmentation is essential for achieving effective radiosensitization.

### 3.2 Key DDR effectors regulating mitochondrial function

ATM and ATR are pivotal kinases and DNA damage sensors responding to IR. A proteomic study identified the phosphorylation substrates of ATM and ATR, revealing that, among 700 proteins induced by IR, 70% are regulated by ATM, uncovering numerous previously unknown DDR-associated signaling networks. Notably, components in the Akt/mTOR pathway, such as including IRS, Akt3, TSC1, 4E-BP1 and p70S6K were found to be involved in DDR (135). The Akt/mTOR signaling pathway is capable of integrating various cellular signals, including growth factors and nutritional status, to not only drive protein synthesis and cell proliferation but also to regulate autophagy, mitochondrial function and energy homeostasis (136). This observation suggests a profound connection between DDR and the Akt/mTOR pathway, potentially shedding light on how DDR regulates mitochondrial function and quality. Moreover, p53, known as the “guardian of the genome”, not only functions as a downstream substrate of ATM but also extensively interacts with Akt/mTOR pathway. For instance, the loss or mutation of p53 frequently leads to the abnormal activation of the Akt/mTOR signaling pathway, a phenomenon that has been validated in various types of cancer (137). Therefore, the ATM/p53/mTOR signaling may constitute a key co-regulatory network linking DDR and mitochondrial. We will detail the specific mechanisms by which ATM/p53/mTOR signaling connects DDR with mitochondrial quality and function (**Figure 2**).

#### 3.2.1 ATM

ATM, a pivotal kinase in DDR, is also crucial for broader cellular homeostasis. It has been observed to be activated in the cytoplasm through a novel mechanism under high concentration of ROS. This activation bypasses autophosphorylation, instead occurring via its original dimer through intermolecular disulfide bonding (138). Further investigation revealed that, compared to cells with nuclear ATM mutations, cells with cytoplasmic ATM deficiencies exhibited increased ROS accumulation and mitochondrial dysfunction (139). Alexander et al. further demonstrated that ROS-induced phosphorylation of cytoplasmic ATM regulates the LKB1/AMPK pathway, inhibiting mTOR and triggering autophagy (140, 141). These findings underscore a critical role for cytosolic ATM in managing oxidative stress and maintaining mitochondrial integrity (**Figure 2**).

Furthermore, ATM activity is crucial for mitochondrial quality. ATM deficiency reduces COX activity and OXPHOS, while ATM overexpression mitigates these effects (142–144). Pathological mitochondrial changes due to ATM dysfunction are evident in human diseases. For instance, Mitochondrial dysfunction is a hallmark of Ataxia Telangiectasia (A-T) and is linked to neurodegenerative disorders, with significant alterations in mtDNA and mitochondrial mass in A-T cells (145). Moreover, ATM and Nbs1 deficient cells exhibit defects of mitophagy under low doses and prolonged radiation, causing abnormal mitochondria accumulation (146). This indicates ATM’s role in mitochondrial quality control through inducing autophagy under IR. Research by Yuehua Wei and colleagues used H1299 ρ^0^ cells (mtDNA depleted) to explore mitochondrial-to-nucleus signaling post-radiation (147). The results demonstrated that, DDR proteins ATM, ATR, and CCNB1 expression increased in H1299 ρ^0^ cells after IR exposure, with differentially genes predominantly enriched in the NF-κB/PI3K/Akt/mTOR pathway.

#### 3.2.2 p53

p53, often hailed as the “guardian of the genome” holds a renowned position in cancer biology. It serves as a critical node in the signaling pathways of ATM and mTOR. ATM directly phosphorylates p53 at Ser 15 or indirectly phosphorylates p53 at Ser 20 to activate and stabilizes p53, thereby inducing cell cycle arrest, promoting DNA repair, and enhancing cell survival (148). Furthermore, ATM-mediated phosphorylation of p53 at Ser46 prioritizes the transcriptional activation of pro-apoptotic genes, such as Fas-R, Bax, Apaf-1, triggering cytochrome c-dependent apoptosis (31, 32). Recent research has also demonstrated that p53 can stimulate the transcription of CDKN1A/p21, PMAIP1/NOXA, and BBC3/PUMA, modulating copper-induced apoptosis (149).

The regulatory role of p53 in autophagy is contingent on its subcellular localization (**Figure 2**). Nuclear p53 promotes autophagy by inducing the transcription of genes such as DRAM, Sestrin1/2, and Bnip3 by activating PTEN, which inhibits the PI3K/Akt/mTOR pathway (141, 150–152). Additionally, nuclear p53 directly activates AMPK, further inhibiting mTOR and promoting autophagy (153). In contrast, cytoplasmic p53 activates mTOR and inhibits autophagy by suppressing AMPK (154). This study discovered that p53 knockout in the cytoplasm elevated the phosphorylation of AMPK and its substrate TSC2 ACCα, while reducing phosphorylation of the mTOR substrate p70S6K, thereby enhancing autophagy. Furthermore, silencing of AMPK or treatment with rapamycin eliminated the observed tendencies in p53-depleted cells. Another study showed that cytoplasmic p53 can prevent Parkin from translocating to damaged mitochondria, thereby inhibiting mitochondrial autophagy (155). Thus, p53’s dual regulation of autophagy is partly mediated by its bidirectional influence on mTOR activity. Radiation-induced autophagy remains significantly affected by p53’s localization and status, thereby impacting cellular radiosensitivity (156, 157).

Research into mitochondrial biogenesis reveals that standard radiotherapy doses activate p53, triggering MDM2-mediated degradation of HIF1α. This process alleviates HIF1α’s inhibition of PGC-1β, thereby enhancing mitochondrial biogenesis and improving mitochondrial quality and function under IR (158). Furthermore, studies show that p53-deficient mice have reduced PGC-1α levels in their skeletal muscles, alongside decreased oxidative respiration, mitochondrial biogenesis, and impaired mitochondrial function, indicating p53’s positive regulatory role in mitochondrial biogenesis (159). However, the regulation of p53 on mitochondrial biogenesis is intricate. Research by Naoyuki Okita et al. revealed that p53 activation inhibits mitochondrial biogenesis in adipocytes, a pattern also seen in chronic lymphocytic leukemia cells (160, 161). This might reflect a compensatory mechanism following the loss of cellular p53. A key mechanism of p53-mediated suppression on mitochondrial biogenesis has been identified in the progression from acute kidney injury to chronic kidney disease. Here, activated p53 binds directly to the PGC-1α promoter in renal tubular epithelial cells, leading to significant transcriptional repression. This results in reduced expression of PGC-1α-dependent targets, such as TFAM and OXPHOS system components (162). Jiuling Li et al. observed that overexpression of p53 in prostate cancer PC3 cells also led to the suppression of PGC-1α expression (163). These findings demonstrate that p53 does not simply promote or inhibit mitochondrial biogenesis, but rather acts as either an “accelerator” or a “brake” depending on the cell type, stress state, and pathological context. This highlights the central and complex role of the p53 network in maintaining cellular energy homeostasis and determining cell fate.

In summary, p53 has been shown to be a crucial pivot in determining cell fate following cellular stress. Its dual nature is evident not only in its dual regulation of autophagy based on its subcellular localization but also in its dual regulation of DDR and mitochondrial biogenesis. In one capacity, p53 can activate cell cycle arrest, facilitate DNA repair, and enhance mitochondrial biogenesis, thereby promoting cell survival following stress exposure. Conversely, p53 can transcriptionally regulate specific genes to induce apoptosis and inhibit mitochondrial biogenesis, ultimately leading to cell death.

#### 3.2.3 mTOR

mTOR, a downstream effector of ATM/p53/mTOR signaling, is pivotal in linking DDR to mitochondrial function. The mTOR is regulated by multiple upstream pathways, with the PI3K/Akt and AMPK pathways being the most crucial (164). The PI3K/Akt pathway plays a pivotal role in protein synthesis and cell proliferation, often triggered by growth factors or nutrition, leading to the activation of mTORC1, a multi-protein complex of mTOR (164). Activated mTORC1 phosphorylates the eukaryotic translation initiation factor 4E-binding protein 1 (4E-BP1) and the p70 ribosomal protein S6 kinase (p70S6K) (165). Phosphorylation of 4E-BP1 releases eIF4E, facilitating mRNA translation, while p70S6K activation phosphorylates ribosomal S6 protein, enhancing translation of mRNAs with a 5’-terminal oligopyrimidine (5’-TOP) motif (165–167). Studies have shown that mTORC1 can regulate the translation of mitochondrial-related proteins encoded by the nuclear genome, thereby activating mitochondrial biogenesis (168). Specifically, mTORC1 overrides the inhibitory effects of 4EBP1 on eIF4E, thereby enhancing the translation of proteins for mitochondrial biogenesis, such as TFAM, PGC-1α, and prohibitin 2 (169, 170).

João F Passos and his research team examined the impact of various DDR activators, such as X-ray exposure and oxidative stress, on mitochondrial biogenesis. They observed that mitochondrial mass increased in a time- and dose-dependent manner following DDR activation, identifying this increase as a downstream event of DDR activation. Gene expression analysis further revealed upregulation of PGC-1β and PGC-1α post-radiation is regulated by the ATM/Akt/mTORC1 signaling pathway (171). As we have previously pointed out, tumor cells adapt to IR-induced DNA damage by enhancing mitochondrial biosynthesis, which is a survival-promoting strategy in response to stress. Preclinical studies have shown that the mTORC1 inhibitor rapamycin can increase the radiosensitivity of tumor cells (172).

mTORC1 suppresses autophagy via multiple pathways, including direct phosphorylation of ULK1 and ATG13 (173–175). Key studies underscore mTORC1’s role in radiation-induced autophagy. Findings indicate that combining mTORC1 inhibitors with radiotherapy enhances autophagy, driving cells into irreversible growth arrest and boosting radiosensitivity. This effect is linked to the prolonged activation of p53, reinforcing the notion that p53 acts as a key upstream modulator of mTOR in response to radiation-induced autophagy (176).

#### 3.2.4 Differences in pathways induced by high-LET and low-LET radiation

Differences in DNA damage create a marked distinction in downstream pathway activation between low and high-LET radiation (177). Multi-omics analyses by D. Story et al. revealed that while both high-LET particles and low-LET radiation activate p53 signaling, high-LET radiation uniquely suppresses genes involved in tumor cell cycling, migration, angiogenesis, and invasion, consistent with its role in metastasis inhibition (178). Additionally, high- and low-LET radiation fundamentally differ in regulating mitochondrial function and radiation-induced bystander effects (RIBE). Low-LET γ-ray-induced RIBE is strictly p53-dependent; for instance, p53 wild-type cells exhibit mitochondrial dysfunction and ROS bursts post-irradiation, leading to micronucleus formation in distal hepatocytes, whereas p53 mutant cells do not. In contrast, high-LET heavy ions (e.g., carbon/iron ions) trigger mitochondrial damage and ROS burst via p53-independent mechanisms, leading to micronucleus formation. This process is insensitive to p53 inhibition but suppressed by mitochondrial inhibitors, highlighting the mitochondrial-ROS axis as central to high-LET RIBE (179). Another research found that carbon ion irradiation at 0.5 Gy increases the expression of mitochondrial respiratory and ATP synthesis proteins in human lymphocytes, highlighting the mitochondrial role in high-LET responses (74). These distinctions influence targeted interventions. Kristina Bannik et al. discovered that ATM inhibitors enhance high-LET α-particle cytotoxicity but are less effective for low-LET X-rays, whereas DNA-PKcs inhibitors show no LET-specificity (180). This result is possibly due to ATM’s broader role including mitochondrial regulation. Consequently, combination radiotherapy regimens should be LET-adapted rather than employing a one-size-fits-all approach.

## 4 Retrograde signaling from mitochondrial dysfunction to the DDR under ionizing radiation

**Figure 3:**
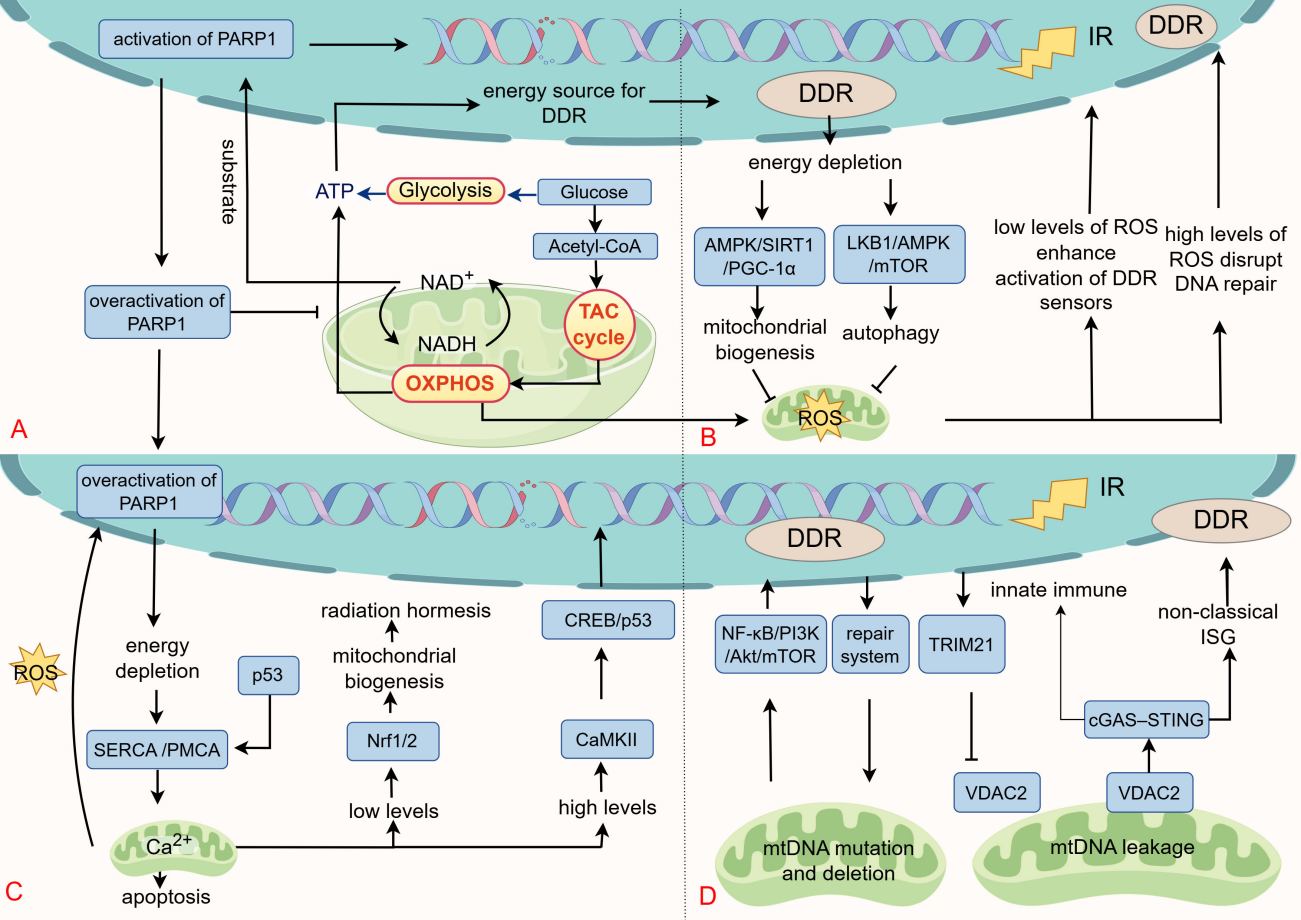
The retrograde signaling from mitochondria to the DDR. **(A)** Mitochondria produce ATP through glycolysis, the TCA cycle, and OXPHOS, providing essential energy for DDR process. Cellular ATP levels are tightly coupled to NAD^+^ availability, a central redox cofactor whose interconversion with NADH drives nutrient catabolism and ATP production. Furthermore, NAD^+^ is a required substrate for PARP1 in the DNA repair to initiate ADP-ribosylation. Pathological overactivation of PARP1 depletes NAD^+^, reduces the NAD^+^/NADH ratio, impairs mitochondrial metabolism, resulting in energetic crisis (ATP depletion) and cell death. **(B)** As secondary products of IR and mitochondria, ROS exert dual regulatory effects on DDR. At low levels, ROS enhances DDR signaling through oxidative modifications (e.g., ATM), supporting cellular survival and repair. In contrast, excessive ROS disrupt DNA repair, promote error-prone pathways, and induce cell death. Energy depletion during DDR stimulates mitochondrial biogenesis and autophagy via the AMPK/SIRT1/PGC1-α and LKB1/AMPK/mTOR axes, restoring metabolic homeostasis and alleviating ROS accumulation. **(C)** IR influences cell fate by modulating Ca²^+^ levels. Low-dose IR induces mild Ca²^+^ signaling, activating Nrf1/2 and promoting radiation hormesis. However, Ca²^+^ overload leads to mitochondrial apoptosis. The DDR pathway regulates Ca²^+^ by modulating Ca²^+^ pump activity under oxidative and radiation stress. **(D)** IR triggers various forms of mtDNA damage, with repair systems preserving mtDNA integrity. mtDNA deletions activate nuclear DDR via the NF-κB/PI3K/Akt/mTOR cascade. Under stress conditions such as radiation, VDAC2-mediated increase in mitochondrial membrane permeability facilitates the leakage of mtDNA into the cytoplasm, activating the cGAS-STING axis and anti-tumor immunity. This process is negatively regulated by TRIM21. Additionally, mtDNA maintains nuclear genome stability through a non-canonical interferon response.

Mitochondria function as both energy producers and critical sensors of the intracellular environment. Under stress conditions like IR, they perceive and integrate signals including ATP, NAD^+^, ROS, Ca²^+^ and mtDNA, transmitting this information to the nucleus via retrograde signaling (181, 182). This communication alters nuclear gene expression and subsequently affects cellular physiology and morphology. This section will analyze the interaction between IR-induced mitochondrial damage, retrograde signaling, and the DDR, and their collective impact on cell fate (**Figure 3**).

### 4.1 ATP and NAD^+^

As cellular “powerhouses” mitochondria generate ATP through tricarboxylic acid cycle (TCA cycle), OXPHOS, and glycolysis, supporting various biological functions (183). In radiotherapy, ATP is vital for DDR enzymatic activities, with its levels directly affecting DDR efficacy (**Figure 3A**). For example, chromatin remodeling complexes, like SWI/SNF, increase DNA damage site accessibility through ATP-dependent mechanisms (184). Helicases, including those in the RecQ family, unwind DNA for repair by ATP hydrolysis (185). Core DDR kinases, such as ATM, ATR, and DNA-PKcs, also rely on ATP for activation (186, 187). Consequently, targeting mitochondrial energy production has emerged as a viable radiosensitization approach. Metformin, originally a glucose-lowering agent, has gained attention in oncology for its ability to suppress complex I of the electron transport chain (ETC), reducing mitochondrial respiration and ATP production, a mechanism with demonstrated antitumor effects (188). Clinical studies indicate that metformin is effective when combined with radiotherapy or chemoradiation in specific cancers (189, 190).

Cellular ATP levels are closely linked to NAD^+^ availability. NAD^+^ serves as a central redox cofactor in major energy-producing pathways, such as the TCA cycle, and OXPHOS, where its interconversion between NADH and NAD^+^ drives catabolic processes and ATP synthesis (**Figure 3A**) (191). Beyond metabolism, NAD^+^ is an essential substrate for PARP1 during the DDR. Upon DNA lesion recognition, PARP1 consumes NAD^+^ to initiate ADP-ribosylation, modifying both itself and adjacent proteins to recruit repair protein, like XRCC1 (192, 193). While PARP1 is known for its role in SSB repair through the BER pathway, it also contributes to DSB repair (HR and NHEJ), nucleotide excision repair, and mismatch repair. Its extensive involvement in genome maintenance has led to its moniker as the “guardian angel of DNA” making it a key target for radiosensitization and synthetic lethal cancer therapies (194). High-affinity PARP inhibitors, like veliparib, rucaparib, niraparib, and olaparib, competitively bind to the NAD^+^ pocket in PARP1’s catalytic domain, inhibiting its activity (195).

PARP1-mediated ADP-ribosylation rapidly depletes cellular NAD^+^, with studies reporting 10-20% depletion within 5–15 minutes of activation, and up to 80% depletion of nuclear NAD^+^ under extreme conditions (196). Appropriate PARP1 activation facilitates DNA repair and enhances radioresistance (194). However, excessive PARP1 activity causes NAD^+^ depletion, reduces the NAD^+^/NADH ratio, and disrupts mitochondrial metabolism (e.g., glycolysis and TCA cycle), leading to ATP deficiency and cell death, a mechanism contributing to neurological injury in degeneration, stroke, and infarction (**Figure 3A**) (197, 198). In such condition, PARP inhibitors exert protective effects and mitigate neural injury (199). Additionally, NAD^+^ also serves as a necessary cofactor for sirtuins, which modulate DNA repair through deacetylation of histone and non-histone proteins (191). For instance, SIRT1 deacetylates p53 to inhibit apoptosis and targets Ku70 to enhance DNA repair (200). Based on these findings, the role of NAD^+^ in post-irradiation DNA repair was examined. Elevating NAD^+^ levels with nicotinamide riboside (NR) after 1 Gy of IR did not alter the efficiency of DSB repair. However, following 5 Gy of IR exposure, NAD^+^-depleted cells still resolved IR-induced DSBs, but with reduced efficiency compared to NAD^+^-replete cells. These findings suggest that NAD^+^ availability limits PARP1 repair effectiveness following high-dose radiation, influencing the cellular damage response (198, 201). Conversely, for radioprotection, administering NAD^+^ precursors like NR maintains NAD^+^ levels, supports mitochondrial function, and mitigates radiation-induced damage (202).

### 4.2 ROS

Irradiation exposure triggers the production of ROS through two primary mechanisms. The first involves the indirect effects of IR, which utilizes the photolysis of water to yield a variety of ROS, hydrated electrons, and secondary free radicals (203). Alternatively, IR-induced mitochondrial dysfunction characterized by mtDNA mutations, decreased OXPHOS, and protein dysregulation, leads to excess O_2_•^-^ and hydrogen peroxide (H_2_O_2_) production in mitochondria (203). This ROS burst further impairs genomic DNA and mitochondria, the latter of which causes ETC dysfunction, dissipates mitochondrial membrane potential (ΔΨm), and opens mPTP, amplifying ROS leakage and perpetuating a cyclic increase in mitochondrial ROS (204–206).

ROS play a complex role in the cellular response to DNA damage. At moderate levels, ROS can serve as amplifiers, enhancing the activation of DDR sensors, while also stimulating mitochondrial quality control to enable adaptive survival in malignancies (207). For instance, H_2_O_2_ directly oxidizes and activates the ATM kinase, establishing a feedforward loop that amplifies the DDR (208). Simultaneously, radiation-induced DDR depletes ATP, increasing the AMP/ATP ratio and activating AMPK. This activation fosters mitochondrial biogenesis via the AMPK/SIRT1/PGC-1α pathway or induces autophagy through the LKB1/AMPK/mTOR axis to clear damaged mitochondria, aiming to restore energy homeostasis and reduce ROS production (209, 210). However, persistently high ROS levels drive cell death and senescence by disrupting repair mechanisms, such as suppressing HR repair through R-loop accumulation and RAD51 dysfunction, and shifting repair toward error-prone NHEJ (**Figure 3B**) (211–213). Vizioli MG and her team have also discovered that excessive ROS activates JNK, disrupting 53BP1 accumulation at damage sites and facilitating the formation of cytoplasmic chromatin fragments under IR (214).

Strategies that modulate ROS by either enhancing their production or inhibiting antioxidant mechanisms significantly boost radiosensitivity. The compound BBT-IR/Se-MN, incorporating diselenide and nitroimidazole groups, increases ROS levels upon X-ray exposure, thereby escalating DNA damage (215). Similarly, 3-methylpyruvate enhances ΔΨm and promotes mitochondrial ROS production, thereby amplifying radiation-induced cell death (216). Inhibiting antioxidant systems also extends ROS activity (217). Notably, the combination of DDR inhibition and ROS activation is particularly effective. For instance, pharmacologic ascorbate (P-AscH^-^) induces H_2_O_2_ accumulation in tumor cells, markedly enhancing the radiosensitizing effects of radiotherapy when combined with the ATM inhibitor KU60019 and other DDR inhibitors, such as ATR inhibitor VE821 and PARP inhibitor veliparib (218).

### 4.3 Ca^2+^

IR influences cell fate by modulating Ca²^+^ levels. ER stress induced by IR can activate IP_3_Rs, triggering ER Ca²^+^ release and elevating cytosolic Ca²^+^ level s (219). Subsequently, mitochondria rapidly absorb Ca²^+^ via the mitochondrial calcium uniporter, elevating mitochondrial matrix Ca²^+^ (220–222). Maintaining mitochondrial Ca²^+^ homeostasis is crucial for signaling and apoptotic regulation (**Figure 3C**). Moderate Ca²^+^ signals induced by low-dose radiation facilitate radiation hormesis though activating Nrf1/2 (223). However, severe Ca²^+^ overload induces apoptosis by increasing mitochondria membrane permeability (222, 224).

There are intricate feedback mechanisms between the DDR and Ca²^+^ homeostasis in the presence of oxidative stress and IR. Overactivation of PARP1 leads to NAD^+^ and ATP depletion, consequently inhibiting Ca²^+^ pump activity (including SERCA and PMCA). This inhibition results in a sustained elevation of cytoplasmic Ca²^+^ levels, triggering mitochondrial Ca²^+^ overload and facilitating necrotic apoptosis through mPTP-mediated pathways (225). Conversely, cytoplasmic p53 localizes to the mitochondria-associated ER, promoting the oxidative modification of SERCA. This modification accelerates Ca²^+^ transport to the mitochondria, leading to mitochondrial Ca²^+^ overload and apoptosis (226). Studies have also demonstrated that IR can induce Ca²^+^-mediated mitotic catastrophes in a Drp1-dependent manner (133). Additionally, Ca²^+^-dependent kinases are crucial in regulating DDR and Ca²^+^ signaling. CaMKII, for instance, enhances cell survival by promoting DDR through the phosphorylation of CREB, regulating p53 phosphorylation, and modulating its transcriptional activity (227). Inhibition of CaMKII with KN93 reduces the clonogenic survival of erythroid leukemia cells treated with IR, suggesting that blocking CaMKII-induced Ca²^+^ overload could be an effective strategy for radiosensitization (228).

### 4.4 mtDNA

Radiation causes diverse mtDNA damage, including point mutations, deletions, and leakage. Low-LET radiation commonly induces a 4977 bp deletion, affecting genes for ATPase, NADPH dehydrogenase complex I, and cytochrome c oxidase (79, 229). In contrast, high-LET radiation predominantly causes mtDNA point mutations, which are key contributors to mitochondrial dysfunction (79, 229). Previous research demonstrated that all deletion of mtDNA could reversely activate DDR proteins through NF-κB/PI3K/Akt/mTOR signaling (**Figure 3D**) (147). Another study also compared the parental human osteosarcoma 143B cells with mtDNA-deficient (Rho^0^ or ρ^0^) 206 cells (derived from 143B cells) under high-energy ultraviolet radiation. The findings showed that, compared to the 143B cells, the Rho^0^206 cells exhibited reduced apoptosis, ΔΨm disruption, and ROS production. This reduction might be attributed to the lack of mtDNA in the cells, leading to low mitochondrial ROS production, and consequently increased resistance to ultraviolet-induced apoptosis (230).

An efficient and economical mtDNA repair system for mtDNA repair is built by cells through the shared usage of nuclear DNA-derived repair proteins (**Figure 3D**) (231). BER serves as the dominant repair pathway for radiation-induced oxidative mtDNA damage. While MMR and DSB repair (HR and NHEJ) might operate in mitochondria, their functions are not fully established (232). Notably, IR can impair repair capacity by downregulating essential repair factors. For instance, studies show that both high-LET proton and low-LET photon irradiation dose-dependently reduce OGG1 (a DNA glycosylase) expression in human astrocytes, thereby diminishing BER-mediated clearance of 8-hydroxy-2-deoxyguanosine (8-OHdG) (233).

Accumulating evidence suggests that under various stressors, including aging, inflammation, tumors, and radio/chemotherapy, endogenous DNA released from the nucleus or mitochondria into the cytosol can trigger innate immune activation via DNA-sensing mechanisms, resulting in ICD (234–238). This response relies on pattern recognition receptors that detect aberrant DNA and initiate downstream signaling, facilitating the clearance of damaged or foreign DNA. The cGAS-STING pathway is central to this process. Upon binding with cytosolic double-stranded DNA, cGAS produces cGAMP, leading to STING activation. STING then traffics to the Golgi, recruits TBK1 and IRF3, and induces type I interferons (IFN-α/β) and immunostimulatory genes (ISGs). This signaling enhances dendritic and cytotoxic T cell activation, strengthening antitumor immunity (239).

Radiotherapy modulates the cGAS-STING pathway by inducing nuclear and mtDNA damage and remodeling the tumor immune microenvironment. Deficiency in 70 - 80% reduces the abscopal antitumor effect of radio-immunotherapy (237). Follow IR, mtDNA release into the cytosol is closely linked to alterations in mitochondrial membrane permeability. In radiotherapy, mtDNA release is linked to VDAC2-regulated mitochondrial membrane permeability and negatively controlled by TRIM21-mediated ubiquitination degradation. This process restrains mtDNA release and subsequent cGAS-STING activation, dampening interferon signaling and antitumor immunity. Inhibition of TRIM21 increases mtDNA release, augments STING pathway activity, promotes dendritic cell maturation and CD8^+^ T cell response, leading to improved radiotherapeutic efficiency and abscopal effects (**Figure 3D**) (240). The role of VDAC2 in mediating mtDNA release is also confirmed in cellular senescence (241).

The fundamental role of autophagy is to preserve cellular homeostasis, which is also reflected in the context of mtDNA-induced antitumor immunity. Research indicates that autophagy can limit the abscopal effect triggered by mtDNA-mediated anti-tumor immunity during radiotherapy. Deficiency in autophagic components, such as ATG5 or ATG7, enhances radiation-induced IFN-α/β secretion and increases radiosensitivity. This effect can be partially reversed by inhibiting cGAS-STING, depleting mtDNA, or blocking mitochondrial membrane permeabilization (242). Although this study suggests that inhibiting autophagy is a promising radiosensitization strategy, its clinical application is limited by the lack of potent and specific inhibitors (242). Of note, mtDNA can initiate non-classical ISG signaling through cGAS-STING in stress conditions, promoting nuclear DNA repair (243). This finding supports a role for mtDNA as a genotoxic stress signal that helps maintain genomic stability.

Consequently, strategies for targeting mtDNA to achieve radiosensitization are multifaceted: 1) Impairment of mtDNA repair (e.g., via OGG1 inhibition) prevents damage clearance, promoting lesion accumulation and mitochondrial dysfunction; 2) Induction of mtDNA release (e.g., by targeting TRIM21 or VDAC) augments cGAS-STING activation, enhancing radiotherapy-induced ICD; 3) Combination with STING agonists or immune checkpoint blockers alleviates tumor-mediated immunosuppression and maximizes antitumor immunity stimulated by dual mtDNA and nuclear DNA damage, culminating in robust radiosensitization and abscopal effects.

## 5 Combination therapies targeting DNA damage and mitochondrial function in radiotherapy

The interplay between the DDR and mitochondrial function offers a rational foundation for innovative combined regimens in radiotherapy. While extensive studies have investigated dual combinations strategy, such as radiotherapy with DDR inhibitors or mitochondrial function inhibition, the three-pronged approach has been underexplored and holds considerable promise. For instance, the synergistic radiosensitization achieved by concurrently using DDR inhibitors and ROS-inducing agents during radiotherapy highlights the potential of this strategy (218). Extending this rationale, combining DDR-targeted strategies with interventions in mitochondrial quality control or energy metabolism may represent a promising synthetic lethality approach for radiosensitization. This effect can be attributed to the critical cellular demand for DDR mechanisms to maintain genomic stability following radiation-induced complex DNA damage, which creates a heightened dependence on ATP and biosynthetic precursors. By simultaneously disrupting the energy production system (via mitochondrial inhibition) and the damage repair machinery (via DDR inhibition), this dual targeting strategy can synergistically induce metabolic and genomic catastrophe, achieving potent synthetic lethality in radiotherapy.

The ENDOLA Phase I/II clinical trial conducted by Max Piffoux and his team of colleagues provides a significant example of this novel combination strategy, despite promoting DNA damage through chemotherapy (244). This study evaluated the safety and preliminary efficacy of an all-oral triple regimen consisting of olaparib (a PARP inhibitor), metronomic cyclophosphamide (a chemotherapy agent), and metformin (a PI3K/Akt/mTOR inhibitor) in patients with recurrent endometrial carcinomas. Noteworthy clinical efficacy was observed in patients who had exhausted multiple treatment lines, with particularly promising outcomes in subgroups lacking a specific molecular signature or harboring TP53 mutations coupled with high genomic instability, where median progression-free survival was 9.1 and 8.6 months, respectively. Approximately 28.5% of endometrial carcinomas are reported to harbor HR deficiency, accompanied by widespread dysregulation of the PI3K/AKT/mTOR pathway across molecular subtypes (244). This signaling axis not only regulates protein synthesis and cell proliferation but also actively promotes mitochondrial biogenesis (164, 169). The inhibitor metformin also exerts anti-tumor effects by targeting mitochondrial energy metabolism and its inhibition of mTOR is linked to energy stress effects and AMPK activation (188). Therefore, the design of this all-oral triple regimen aligns with the principle of synthetic lethality. Cyclophosphamide causes DNA damage, heightening dependence on repair mechanisms. Olaparib impedes SSB repair, converting SSBs into lethal DSBs. And metformin inhibits the PI3K/Akt/mTOR pathway and mitochondrial function, disrupting compensatory proliferation and energy supply for DDR.

## 6 Conclusions and perspectives

Radiotherapy triggers a highly integrated cellular stress response system by inducing DNA damage and mitochondrial dysfunction. IR directly causes nuclear DNA damage, particularly complex DSBs, activating the DDR and initiating cell cycle arrest, repair, or apoptosis. Simultaneously, mitochondria perceive radiation stress through morphological alterations, functional changes (such as loss of ATP and ROS burst), and mtDNA damage, thereby activating quality control mechanisms like mitophagy, biogenesis, fusion, and fission. This dual sensing mechanism ensures a multi-layered cellular response to radiation. Information integration between these systems relies on a dynamic, bidirectional signaling network. Nuclear DDR signals (e.g., ATM, p53, mTOR) can downstream regulate mitochondrial biogenesis, dynamics, and autophagy; meanwhile, mitochondria upstream modulate nuclear gene expression, DDR efficiency, and even immune responses (e.g., via cGAS-STING activation) through the release of signaling molecules such as ATP, ROS, Ca²^+^, and mtDNA. Key molecules including ATM, p53, mTOR, and AMPK serve as critical integrators in this nucleus-mitochondria crosstalk. These complex interactions ultimately determine cell fate: promoting survival through enhanced biogenesis and autophagic clearance of damaged mitochondria, or driving cell death, such as apoptosis, ferroptosis, or ICD via membrane depolarization, ROS outburst, mtDNA release, ATP depletion and cGAS-STING activation. Consequently, targeting these interactive nodes (e.g., combining DDR inhibitors with mitochondrial modulators) has been shown to significantly enhance radiosensitivity, holding considerable promise for combination therapy.

However, the rational design of combination radiotherapy strategies necessitates a systems-level view of pathway interactions to precisely direct cellular outcomes. Using autophagy as an example, this process is embedded in an interactive circuitry, wherein selective pathway activation yields distinct effects. Activation of mTOR promotes protein synthesis and mitochondrial biogenesis while inhibiting autophagy, often leading to radioresistance. Nuclear p53 activation induces cell cycle arrest, apoptosis, and autophagy, while cytoplasmic p53 suppresses autophagic activity. Low-dose radiation may promote adaptive pro-survival autophagy, whereas high-dose exposure can trigger lethal autophagy or apoptosis. Autophagy is thus not a binary switch but a context-shaded process shaped by mTOR activity, p53 compartmentalization, DDR status, mitochondrial integrity, ROS levels, bioenergetic state, and radiation quality. The dynamic interplay among these elements determines autophagy toward survival or death. Decoding this network supports informed combination therapy; for example, mTOR inhibition with rapamycin in p53 wild-type tumors could suppress proliferation and enhance p53-mediated autophagy and apoptosis (245).

In conclusion, combinatorial regimens incorporating DDR inhibitors and mitochondrial inhibitors possess strong and feasible clinical potential. Co-inhibition imposes dual stress that overwhelms cellular compensatory mechanisms, markedly augmenting radiotherapy effectiveness and circumventing limitations of single-agent targeting, including acquired resistance. Moreover, approved drugs like metformin and the PARP inhibitor olaparib offer expedited translation to clinical trials due to their established safety profiles. Clinical evidence has supported the efficacy of metformin in combination with radiotherapy in some cancers (189, 190). Importantly, genetic sequencing and mitochondrial signature analysis prior to radiotherapy, and patient classification based on DDR deficiency status and mitochondrial phenotype, can optimize personalized radiation therapy strategies.
